# Diabetic endothelial microangiopathy and pulmonary dysfunction

**DOI:** 10.3389/fendo.2023.1073878

**Published:** 2023-03-21

**Authors:** Lanlan Zhang, Faming Jiang, Yingying Xie, Yan Mo, Xin Zhang, Chuntao Liu

**Affiliations:** ^1^ Department of Respiratory and Critical Care Medicine, Division of Pulmonary Diseases, State Key Laboratory of Biotherapy, West China Hospital of Sichuan University, Chengdu, China; ^2^ Department of Nephrology, Seventh Affiliated Hospital, Sun Yat-sen University, Shenzhen, China; ^3^ Department of Neurology Medicine, The Aviation Industry Corporation of China (AVIC) 363 Hospital, Chengdu, China; ^4^ Department of Gastroenterology, West China Hospital of Sichuan University, Chengdu, China

**Keywords:** endothelial cells, COVID-19, asthma, COPD, type-2 diabetes

## Abstract

Type 2 diabetes mellitus (T2DM) is a widespread metabolic condition with a high global morbidity and mortality rate that affects the whole body. Their primary consequences are mostly caused by the macrovascular and microvascular bed degradation brought on by metabolic, hemodynamic, and inflammatory variables. However, research in recent years has expanded the target organ in T2DM to include the lung. Inflammatory lung diseases also impose a severe financial burden on global healthcare. T2DM has long been recognized as a significant comorbidity that influences the course of various respiratory disorders and their disease progress. The pathogenesis of the glycemic metabolic problem and endothelial microangiopathy of the respiratory disorders have garnered more attention lately, indicating that the two ailments have a shared history. This review aims to outline the connection between T2DM related endothelial cell dysfunction and concomitant respiratory diseases, including Coronavirus disease 2019 (COVID-19), asthma, chronic obstructive pulmonary disease (COPD) and idiopathic pulmonary fibrosis (IPF).

## Introduction

Monitoring other diseases associated with diabetes is necessary for patients with Type 2 diabetes mellitus (T2DM). Vascular diseases such as coronary artery disease, cerebrovascular accidents, retinopathy, nephropathy, and neuropathy are causes of morbidity and mortality in diabetes (1). Also, diabetic patients are more prone to asthma, chronic obstructive pulmonary disease (COPD), obstructive sleep apnea syndrome (OSA), acute lung injury, and respiratory infections (2). In addition, many studies have shown that various metabolic pathologies accompany respiratory diseases, and the pathophysiological mechanisms that determine the major degenerative complications of diabetes may also contribute to the leading cause of lung function deficits (3, 4).

Studies have shown that endothelial dysfunction largely influences diabetic vasculopathy’s pathophysiology. Even in the early stages of diabetic microangiopathy, endothelial dysfunction is already present (5–7). Although it is unclear whether endothelial dysfunction is a feature of diabetes itself or whether other factors are necessary to cause endothelial dysfunction, given the presence of diabetes, it is believed that endothelial dysfunction is the hallmark stage of diabetes (8). Many factors contribute to the emergence of endothelial dysfunction in diabetes. Activation of protein kinase C (*PKC*), increased expression of transforming growth factor-beta (*TGF-β*) and vascular endothelial growth factor (*VEGF*), non-enzymatic glycation, oxidative stress, activation of the coagulation cascade, increased expression of tumor necrosis factor-alpha (*TNF-α*), high levels of insulin and insulin precursor molecules, and hyperglycemic pseudohypoxia are a few common hypotheses. These factors may all contribute to endothelial dysfunction However, in the situations of diabetes and lung disease, the importance of these proposed pathways has not been evaluated (5).

Given that the lungs are anatomically covered in a significant number of vascular endothelial cells and that this increases the likelihood that endothelial cell damage brought on by diabetic lesions will result in lung disease, endothelial cells play a special role in “connecting” diabetes and lung disease. The discovery of abnormal pulmonary function in some diabetic patients raise the possibility that the lung should be viewed as a “target organ” for diabetes (8, 9). Endothelial dysfunction in chronic lung diseases has been gradually increasing in recent years, especially in Coronavirus disease 2019 (COVID-19) and idiopathic pulmonary fibrosis (IPF), COPD, and asthma, while endothelial dysfunction in diabetes leading to acute or chronic airway diseases of the respiratory is almost rarely studied (10–13). The primary purpose of this review is to examine the mechanisms of action of the vascular endothelial cells as a shared target in the co-morbidity of T2DM and respiratory disease (COVID-19,COPD, asthma, and IPF) as well as the effects of T2DM treatment on respiratory disease *via* the vascular endothelial cells.

## Potential mechanisms of diabetes related lung disease

The traditional role of blood vessels is to carry oxygen and vital nutrients to other tissues. The primary core lesion cells in most of these vascular disorders are endothelial cells. Long believed to be only controlled by angiogenic growth factors like *VEGF* and other signals like Notch, research indicates that the metabolic switch in endothelial cells also has an impact on the angiogenic switch (14). The vessel wall is impacted by the metabolic environment of T2DM, which includes endothelial dysfunction, platelet overactivity, oxidative stress, and inflammation, as well as insulin resistance, hyperglycemia, and the formation of excess free fatty acids and other metabolic abnormalities. Vasoconstriction is further intensified, and thrombosis is expedited when these mechanisms are active. The endothelial dysfunction driven on by diabetes is a critical early stage that cannot be ignored in the development of vascular issues. Reduced nitric oxide (NO) release, increased oxidative stress, increased generation of inflammatory agents, aberrant angiogenesis, and impaired endothelium repair are the typical symptoms of endothelial dysfunction in microvascular issues (15).

In clinical practice, abnormal pulmonary function is observed in some diabetic patients; the most consistent abnormalities are reduced lung volume, pulmonary elastic recoil, and pulmonary diffusion impairment in both young and adult diabetic subjects, which are caused by decreased capillary blood volume in the adult group (16, 17). The existence of pulmonary microangiopathy is suggested by the histological data, which also shows thickening of the basal lamina of the pulmonary capillaries (18). The most frequent pathogenetic explanation for mechanical pulmonary dysfunction in diabetic subjects is non-enzymatic glycosylation-induced pulmonary connective tissue changes, while the most plausible explanation for impaired pulmonary microangiopathy in these patients is the presence of underlying pulmonary microangiopathy; abnormal endothelial cell function is another frequent mechanism (18, 19). It has been reported in the literature that common pathogenetic mechanisms exist between diabetes and respiratory disorders and that these mechanisms may play a significant role in the diabetes-induced decline in lung function (**Table 1**). However, further research is presently needed to determine the importance of endothelial cell dysfunction for respiratory disease.

**Table 1:** T2DM pathophysiology shared by COVID-19, asthma, COPD and IPF.

Common mechanisms		Study			COVID-19	Study			T2DM		
Activation of Protein Kinase C		Huang, Changbai et al. (20)			In *ACE2*-expressing A549 cells, PKC inhibitors prevented the reproduction of wild-type SARS-CoV-2	Beeson, Mary et al. (21)			Reduced capacity of *PIP3* to directly activate *aPKCs* and impaired insulin receptor substrate (IRS)-1-dependent PI 3-kinase activation		
Increased expression of tumor necrosis factor		Hsu, Ren-Jun et al. (22)			High systemic TNF- levels were linked to respiratory distress syndrome, lower survival, and pulmonary dysfunction in severe COVID-19 cases	Makowski, Lena-Maria et al. (23)			T2DM patients’ monocytes have an enhanced migratory response to low *TGF-1* concentrations		
Oxidative Stress		Suhail, Shanzay et al. (24)			The *ACE2* receptor is essential for reducing oxidative stress	Apostolova, Nadezda et al. (25)			Key metabolic processes are disrupted (*AMPK* and *mTORC1*)		
Common mechanisms		Study			COPD	Study			T2DM		
Oxidative stress		Barnes, Peter J (26).			Reactive oxygen species (*ROS*) cause endogenous antioxidant defenses to become compromised and/or overpowered, which leads to oxidative stress	Apostolova, Nadezda et al. (25)			Interfere with major metabolic pathways (*AMPK* and *mTORC1*)		
Increased expression of tumor necrosis factor		Feng, Qiong et al. (27)			*TNF-α* - knockdown may reduce *MAPK* pathway activation while increasing *SOCS3/TRAF1* expression.	Makowski, Lena-Maria et al. (23)			High glucose levels induced soluble(s) *VEGFR1* expression.		
Common mechanisms		Study			Asthma	Study			T2DM		
Activation of protein kinase C		Lu, Yiwen et al. (28)			Eosinophil peroxidase potentiates the *CCDC25-ILK-PKC-CRTC1* pathway, which is used by EETs to activate pulmonary neuroendocrine cells.	Beeson, Mary et al. (21)			Insulin increased muscle *aPKC* activity in control participants by threefold, which was due to diminished IRS-1-dependent PI 3-kinase activation and reduced capacity of PIP3 to directly activate *aPKCs*.		
Oxidative stress		Michaeloudes, Charalambos et al. (29)			Excessive generation of *ROS* brought on by immune cells invading, especially eosinophils and neutrophils	Apostolova, Nadezda et al. (25)			Interfere with major metabolic pathways (*AMPK* and *mTORC1*)		
Common mechanisms		Study			IPF	Study			T2DM		
Genetic predisposition		Stock, Carmel J et al. (30)			*MUC5B* variant increases risk of IPF	Chen, Guanjie et al. (31)			Dysregulated *MUC5B* expression may be involved in the pathogenesis of T2DM	
Increased expression of TGF-β		Chanda et al. (32); Joannes et al. (33); Konigshoff et al. (34); Selman et al. (35)			activation of *TGF-β* pathways in IPF	Zhou, T. et al. (36)			High levels of *TGF-β1* are associated with susceptibility to T2DM	
Oxidative Stress		Gonzalez-Gonzalez, Francisco J et al. (37)			*ROS* play a signaling role to enhance *TGF-β* signaling and promote fibrosis	Apostolova, Nadezda et al. (25)			Key metabolic processes are disrupted (*AMPK* and *mTORC1*)	
Activation of protein kinase C		Wang, Jun et al. (38)			Protein Kinase C δ (*PKCδ*) Attenuates Bleomycin Induced Pulmonary Fibrosis *via* Inhibiting *NF-κB* Signaling Pathwayb	Beeson, Mary et al. (6)			Reduced capacity of *PIP3* to directly activate aPKCs and impaired insulin receptor substrate (IRS)-1-dependent PI 3-kinase activation

**Table 1** By identifying common signaling pathways between diabetes and pulmonary dysfunction in respiratory diseases, we sought to identify the mechanisms of lung function deterioration in diabetes combined with COVID-19, asthma, COPD, and IPF. The common mechanisms of COVID-19 in combination with diabetes mellitus are protein kinase C, tumor necrosis factor, and oxidative stress. The common mechanisms of COPD are oxidative stress and tumor necrosis factor. The common mechanisms associated with asthma are protein kinase C and oxidative stress. ACE2, angiotensin converting enzyme-2; SARS-CoV-2, severe acute respiratory syndrome coronavirus 2; PIP3, Phosphatidylinositol-3,4,5-triphosphate; aPKC, Atypical protein kinase C; TGF-β1, Transforming growth factor beta 1; AMPK, AMP-activated protein kinase; mTORC1, mammalian target of rapamycin complex 1; SOCS3, Suppressor Of Cytokine Signaling 3; MAPK, Mitogen−activated protein kinase; TRAF1,TNF Receptor Associated Factor 1; CCDC25, Coiled-coil domain containing 25; ILK, Integrin-linked kinase; PKC, Protein kinase C; CRTC1,CREB-regulated transcription coactivator 1; MUC5B, Mucin 5B.

## Common etiology of T2DM related endothelial microangiopathy

**Figure 1:**
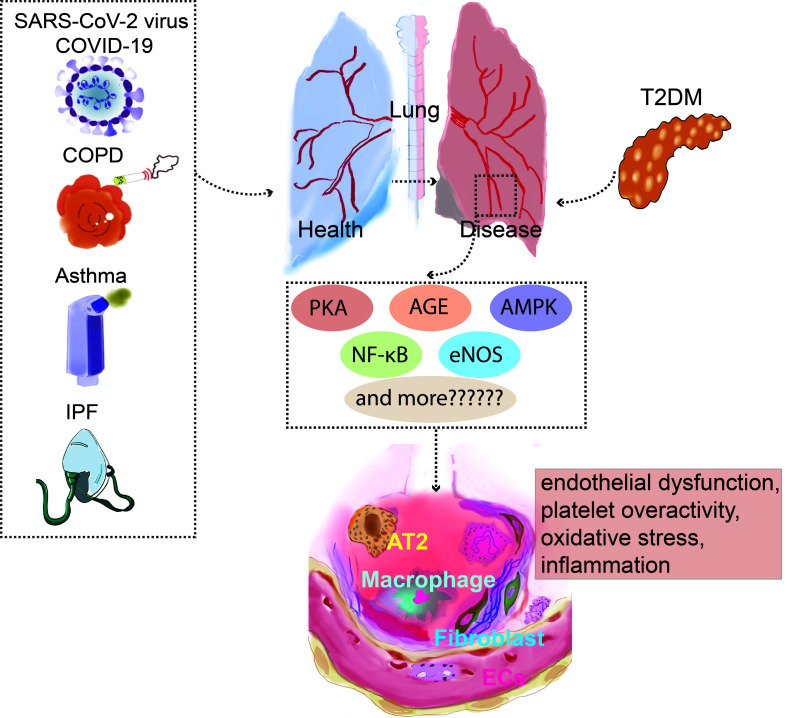
*PKA,AGE,AMPK*, NF-κB*,eNOS* are presented as an example for a list of prospective therapeutic targets that diabetes and pulmonary diseases potentially explore through vascular endothelial cells. NF-κB, Nuclear factor kappa B; AT2, Alveolar Type II Cells; COPD, Chronic obstructive pulmonary disease; *PKC*, Protein kinase C; COVID-19, Coronavirus disease 2019; eNOS, Endothelial NO synthase; *AMPK*, AMP-activated protein kinase.

Fewer research has evaluated the processes of endothelial cells as a shared target for diabetes and respiratory disorders. Different organ systems, even vascular beds in the same area of a vascular bed exhibit considerable variability in endothelial cell responses. In addition to the variances, there are shared signaling pathways. The research of Nitric oxide (NO) has focused particularly on prostacyclin and NO, two key byproducts of endothelial cells that are crucial for managing vascular homeostasis. Endothelial NO synthase (eNOS), which is crucial for the regulation of endothelial function, produces NO *via* an enzymatic process. Studies in models of T2DM have shown that aberrant *NADPH* oxidases (NOX) activation results in endothelial dysfunction and eNOS (39). Recent studies have shown that there is a common pathogenesis in targeting vascular endothelial cells for the treatment of diabetes and pulmonary diseases. Glucagon-like peptide-1 (*GLP-1*), for instance, are extensively used in the treatment of diabetes, and the research shows that it relates to the function of the vascular endothelial cells. *GLP-1* decreased reactive oxygen species-induced senescence in human umbilical vein endothelial cells (HUVECs) in a receptor-dependent manner that included downstream Protein kinase cAMP-dependent (*PKA*) signaling and the activation of antioxidant genes (40). Studies conducted *in vitro* on HUVECs revealed that GLP-1 receptor agonists (GLP-1RAs) decreased reactive oxygen species-induced senescence in a receptor-dependent manner that included downstream *PKA* signaling and activation of antioxidant genes (41). Advanced glycation end products (AGEs) increased endothelial cells, however GLP-1-RAs suppressed these endothelial cells by reducing AGE receptor (*RAGE*) expression and upregulating *VCAM-1* mRNA levels (42). Inhibiting *PKC-α*, *NADPH* oxidase, *NF-κB* signaling, and upregulating anti-inflammatory and antioxidant enzymes, GLP-1RAs significantly reduced inflammation and had antioxidant effects on endothelial cells (43). GLP-1RAs significantly reduced inflammation in human aortic endothelial cells. They also showed that it could raise intracellular Ca^2+^ and activate Calcium/calmodulin-dependent protein kinase kinase (*CAMKK*), which in turn could activate *AMPK (* 44). Endothelin-1, which is also present in endothelial cells, is inhibited by GLP-1RAs *via* preventing the activation of nuclear factor kappa B (45). Common diseases of pulmonary dysfunction, such as COVID-19, asthma, COPD, IPF, and T2DM, have abnormal vascular endothelial cell function, and diabetes drug targets are commonly expressed in the vascular endothelial cells (**Figure 1**); However, in addition to common signaling pathways, lung diseases share their specific targets with T2DM when pulmonary vascular endothelial cells are used as common targets.

## The unique pathophysiology of pulmonary dysfunction and endothelial microangiopathy driven by T2DM

### COVID-19 and T2DM

**Figure 2:**
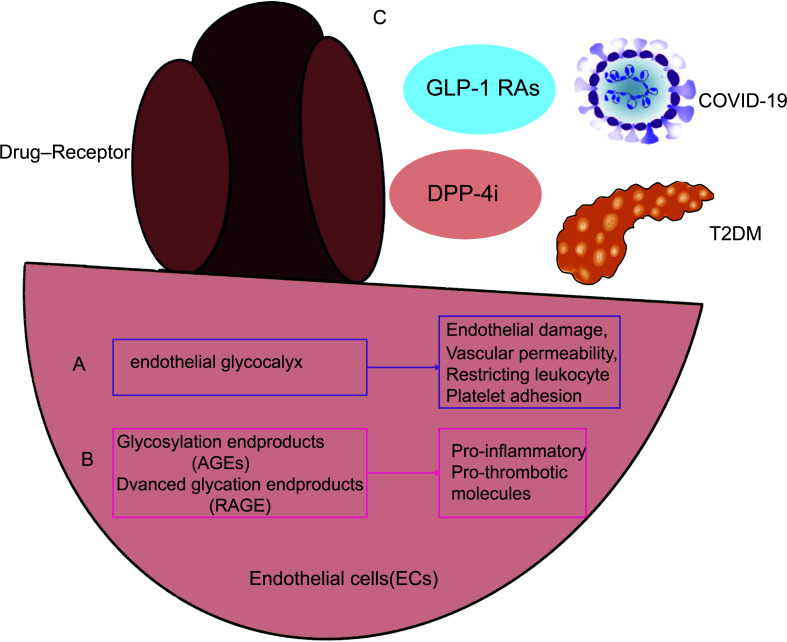
illustrates how endothelial cell damage from T2DM and COVID-19 infection are related. As a result, T2DM paired with COVID-19 infection may trigger the same signaling pathways and processes via endothelial cells, including endothelial glycocalyx **(A)**, RAGE, and AGEs **(B)**. Additionally, maintaining good management of diabetes and using glucose-lowering medicines **(C)** by acting on endothelial cells may lessen the likelihood that COVID-19 infection may result in decreased pulmonary function. GLP-1 RAs, GLP-1 receptor agonists; DPP-4i, Dipeptidyl peptidase 4 inhibitors.

A few of the several potential causes of COVID-19 in combination with diabetes are increased inflammatory storms, immunocompromised states, disturbance of glucose homeostasis, hypercoagulable state, alveolar hyperpermeability, and vascular endothelial injury. The characteristics of patients with severe COVID-19 may be explained by the endothelial glycocalyx (**Figure 2A**). According to study, acquired *Hpa-2* deficiency may be a possible causative factor in patients with severe COVID-19 with endothelial damage involving the integrity of the glycocalyx (46). A key element of vascular integrity and cardiovascular homeostasis is the endothelial glycocalyx (EG), which covers the apical surface of endothelial cells and floats into the lumen of the channel. Most EG-related functions include separating blood from the endothelium, regulating vascular permeability, restricting leukocyte and platelet adhesion, and improving the endothelial responsiveness to flow fluctuations through mechanosensing. Recent research findings indicate a more inventive strategy for maintaining EG levels in T2DM therapy (47). A substantial part of the glycocalyx that constitutes the vascular wall and endothelium glomerular permeability barrier is hyaluronic acid (HA). It is known that endocytosed hyaluronidase1 (*HYAL1*) breaks down HA into tiny pieces in a variety of cell types, including endothelial cells. Diabetes-related endothelium and glycocalyx dysfunction is facilitated by *HYAL1*. A novel treatment strategy to stop the vascular consequences of diabetes may use *HYAL1* inhibitors (48, 49).

The vascular EG may provide an explanation for the traits of COVID-19 patients who are severely unwell. The vascular EG is disturbed by inflammation brought on by SARS-CoV-2 infection, as well as in people with hypertension, obesity, diabetes, cardiovascular disease, and current smokers. The elderly may be more susceptible to SARS-CoV-2 infection than younger people, and males are more likely to get the virus than females (11). The pulmonary interstitial develops aberrant shadows (multiple speckled shadows with a ground glass interstitial appearance) as a result of microvascular leaks caused by vascular glycocalyx failure. It also triggers the coagulation cascade, which may result in multi-organ failure and thrombosis. Notably, the virus that causes COVID-19, SARS-CoV-2, interacts to angiotensin converting enzyme-2(*ACE2*), which is abundantly present in human lung and small intestine epithelial cells as well as vascular endothelial cells and arterial smooth muscle cells. EG abnormalities may be linked to persistent inflammation during endotoxemia in a diabetic mouse model (47). According to Lambadiari et al., persistent cardiovascular symptoms after 4 months were associated with markers of EG and vascular function (50).

Endothelial markers of COVID-19 invasive ventilation increase in correlation with the outcome of vascular damage, whereas endothelial injury indicators upregulate later (51). *RAGE* is abundantly expressed in the membranes and cytoplasm of pneumocytes in the lung, where it interacts with AGEs in patients with combined diabetes in COVID-19, activating downstream signaling pathways and inducing an inflammatory response (**Figure 2B**). In consequence of this, vascular wall cells generate more cytokines and have greater endothelial permeability (52). *RAGE* mediates cell migration and the activation of pro-inflammatory and pro-thrombotic molecules on cells including endothelial cells by interacting with ligand-*RAGE* on those cells (53). Targeting this mechanism, particularly in individuals with combined Diabetes, may help prevent cytokine storm and thrombotic symptoms related to dysregulated immunological response to SARS-CoV-2 infection (54).

The anti-inflammatory features of incretin-based treatments, diabetes treatment medications were suggested to improve the prognosis of COVID-19 (**Figure 2C**). Despite the older age and typically more severe disease of DPP4i users, the evidence demonstrates that pre-morbid usage of GLP1-RA is linked with decreased mortality and other deleterious outcomes compared to DPP-4i use in COVID-19 patients (55). According to study, DPP-4i users had an increased adjusted risk ratio for 30-day mortality (56). Patients with T2DM with COVID-19 have a reduced risk of death if they take metformin and sulfonylureas. However, mortality is higher in T2DM patients with COVID-19 and on insulin (57, 58). However, there are also reports in the literature that treatment with sulfonylureas is not associated with mortality (59).

In conclusion, individuals with COVID-19 infection, in addition to diabetes, have worse lung function than those with diabetes alone. One of the key mechanisms for this is the activation of endothelial cells, including endothelial glycocalyx, *RAGE*, and *AGEs*. Treatment for diabetes may also delay the decline in lung function brought on by COVID-19 infection since the medications used to manage diabetes may also have effects on the lungs, delaying the progression of pulmonary disease.

### COPD and T2DM

Patients with COPD are more likely than the general population to have T2DM.Additionally, T2DM patients are more likely than non-diabetic individuals to have concomitant COPD (60, 61). The increasing incidence of diabetes in COPD has been linked to several variables, such as rising obesity, declining physical activity, rising smoking, exposure to corticosteroids, disease-related inflammation, oxidative stress, and hypoxia. Smoking, inflammation, or obesity by individually, however, do not completely explain the link between T2DM and reduced lung function. Despite controlling for other risk variables, airflow restriction also seems to be an independent predictor of death in persons with T2DM.

**Figure 3:**
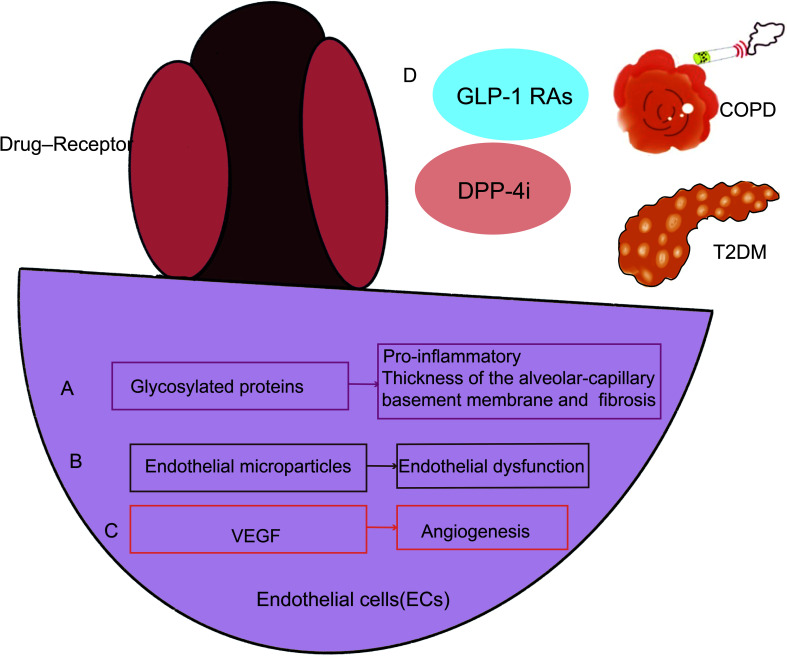
In endothelial cells, COPD and T2DM create a similar pathogenic pathway. Endothelial cells in T2DM may impact how a lung disease develops in the end. Endothelial cells become dysfunctional in patients who have T2DM and COPD because of the accumulation of glycosylated proteins **(A)**, a rise in endothelial microparticles (EMPs) **(B)**, and an increase in VEGF **(C)**. GLP-1 RAs, GLP-1 RAs and DPP-4i can treat COPD and diabetes simultaneously **(D)**. GLP-1 RAs, GLP-1 receptor agonists; DPP-4i, Dipeptidyl peptidase 4 inhibitors; VEGF, Vascular endothelial growth factor.

Among the hypothesized mechanisms to contribute for the decline in lung function associated with diabetes include microangiopathy of the alveolar capillaries and pulmonary arterioles, chronic low-grade tissue inflammation, autonomic neuropathy affecting the respiratory muscles, and loss of elastic recoil secondary to collagen glycosylation of lung parenchyma (62–65). The accumulation of glycosylated proteins, which have pro-inflammatory tendencies, in persistent hyperglycemia is the underlying cause of diabetes’ microvascular sequelae. The interstitium widened and the alveolar gap closed as a result of increased pulmonary vascular permeability, mononuclear cell infiltration, cell proliferation, hypertrophy of interstitial cells, and accelerated fibrosis, as depicted in studies on the lungs of diabetic rats (66, 67). In human autopsies (68) and trans-bronchial biopsy investigations (69), alterations in the thickness of the alveolar-capillary basement membrane and nodular fibrosis in the alveolar walls have been linked to diabetes. Given the alveolar-capillary basement membrane has undergone fibrotic modifications and an enlarged interstitium inhibits lung expansion, these changes may clinically translate to lower spirometry scores and decreased diffusion (**Figure 3A**).

Clinical data suggest that the vascular area in the airways of COPD patients is increased and may lead to narrowing of the airways (70). Endothelial dysfunction is defined as disturbed endothelium-dependent vasodilation. Endothelial microparticles (EMPs) in blood can also be used as a measurement of endothelial dysfunction. Endothelial dysfunction correlates with the severity of COPD and is associated with forced expiratory volume in 1 second (FEV1) (71–73). EMPs levels are elevated in patients with frequently worsening COPD (74) and also predict a rapid decline in patient FEV1 (75). EMPs are positively correlated with the severity of emphysema in COPD patients, again suggesting that endothelial cells may be a potential mechanism for emphysema (75). EMPs are also associated with T2DM (76–81) (**Figure 3B**).

*VEGF* is a highly specific growth factor that targets endothelial cells and is produced in response to hypoxia (82). *VEGF* levels may be reduced in such patients because the major transcription factor of *VEGF*, hypoxia-inducible factor 1α (*HIF-1α*), which mediates cellular and systemic responses to hypoxia and binds to the hypoxia response element (HRE) on *VEGF (* 83). T2DM-associated endothelial angiogenesis and rising *HIF-1* levels are tightly connected. VEGF expression was reduced, and angiogenesis was prevented by *HIF-1a* inhibition. New treatment options for diabetic retinopathy were revealed by *VEGF* of endothelial cells (84–89).Further exploration of molecular mechanisms of COPD suggests that increased bronchial vascular distribution is associated with higher cellular expression of *VEGF-A* (90, 91). Levels of *HIF-1α* and *VEGF* may correlate with predicted FEV1 percentages in COPD patients (83, 92) (82). Excessive stimulation, including cigarette smoke (55), hypoxia (93) and cytokines) (94) increase *VEGF-A* production (95). Perfusion of isolated lungs under hypoxic conditions increases tissue *VEGF-A* and *VEGFR1* (96, 97). The above studies confirm that endothelial cells initiate and participate in the pathogenesis of COPD, T2DM which induces vascular inflammation with proinflammatory, and remodeling activities (98). As research progresses, there is increasing evidence that molecular biological mechanisms such as endothelial cells play an important role in regulating endothelial cell dysregulation in COPD and T2DM (**Figure 3C**).

GLP-1RAs may enhance endothelial function *via* a variety of pathways, some of which are independent of insulin signaling or glucose homeostasis. GLP-1RAs is a potential novel therapeutic target for controlling COPD and T2DM together (69–71). *GLP-1* has anti-inflammatory and surfactant-releasing effects, so studies showed that GLP-1Ras reduce the frequency of acute exacerbations by decreasing their severity and that GLP-1RAs may have therapeutic potential for the treatment of COPD (99–102). Endothelial cells express DPP-4, which has a broad variety of biological roles in glucose metabolism, cancer biology, and immunological control. DPP-4i is detrimental in respiratory diseases. Slowly but surely, its biological processes, important molecular pathways, connections, and linkages are being revealed. Respiratory diseases may be affected significantly by DPP-4i and subsequent development may be regulated (103) (**Figure 3D**). As diabetes may lead to an increased prevalence of COPD, and in turn COPD may lead to an increase in diabetes, future research should take advantage of the mechanisms of endothelial cell dysfunction to improve clinical outcomes in patients with diabetes combined with COPD.

### Asthma and T2DM

Both early-onset diabetes and asthma are becoming more common, and adolescents who have active asthma have an increased chance of developing T2DM (104). A prospective cohort of 38,570 women, both asthma and COPD were separately and independently linked to a higher risk of developing T2DM, suggesting that chronic airway inflammation may play a role in the development of diabetes (105). In a significant nested case-control research, patients with asthma or COPD who used inhaled corticosteroids (ICS) saw a 34% higher incidence of diabetes mellitus during 5.5 years of follow-up compared to age-matched controls who did not get ICS (106). The findings are consistent with the notion that metabolic traits linked to prediabetes and diabetes, such as metabolic syndrome and insulin resistance, might affect asthma morbidity (107). Patients with concurrent DM who were hospitalized for asthma had longer hospital stays, higher costs, and a higher risk of readmission. In patients with coexisting DM and asthma, interventions are urgently required to lower the risk of hospital admission and readmission (108, 109).

**Figure 4:**
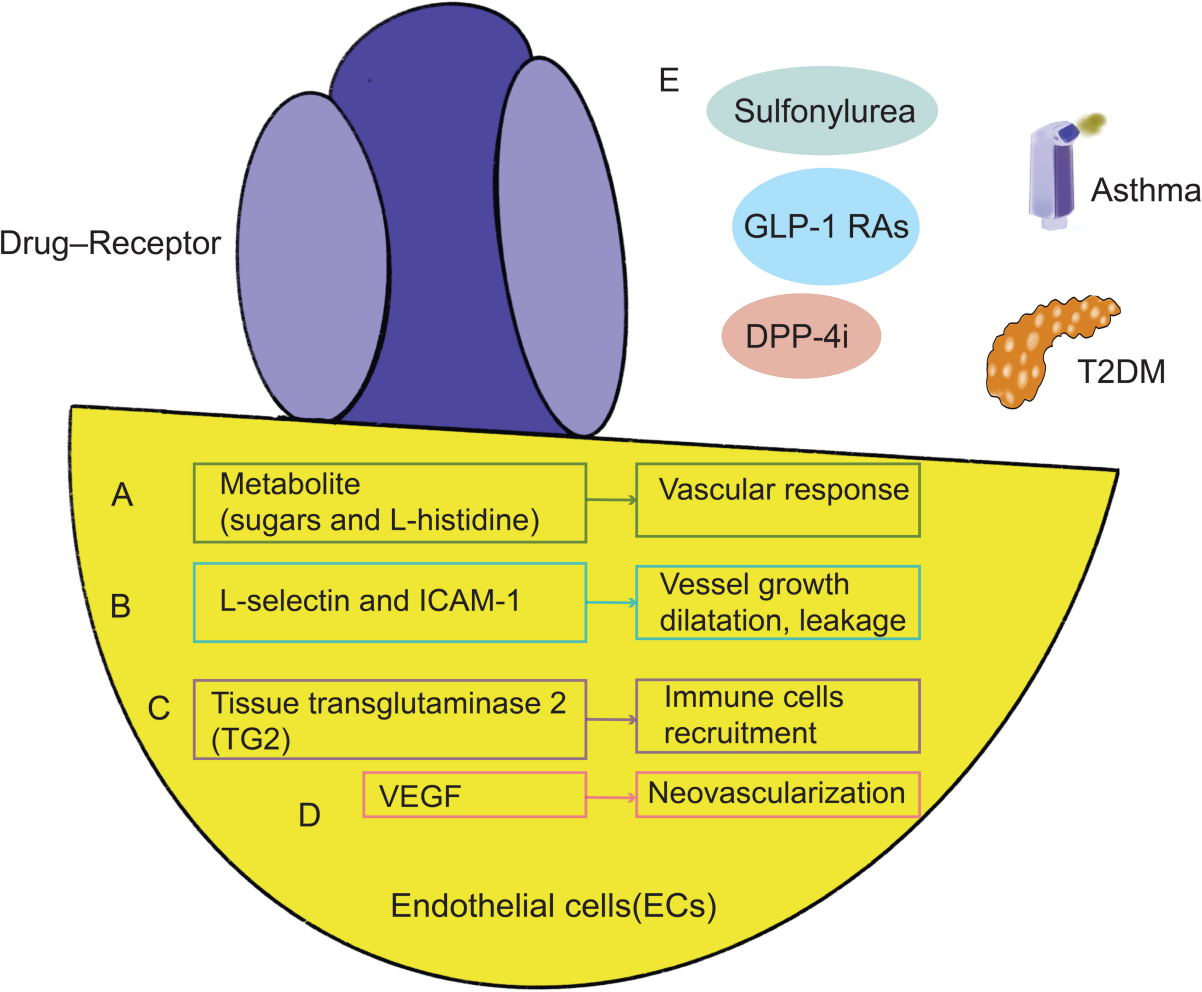
Mechanisms shared by asthma and T2DM in endothelial cells. Metabolite **(A)**, L-selectin, ICAM-1 **(B)**, tissue transglutaminase 2 (TG2) **(C)**, and VEGF **(D)** are all intimately connected to and have an impact on each other in endothelial cells, which may represent a common mechanism of lung function decline. GLP-1 RAs targeting endothelial cells, DPP-4i and other drugs can treat asthma and diabetes **(E)**. GLP-1 RAs, GLP-1 receptor agonists; DPP-4i, Dipeptidyl peptidase 4 inhibitors; VEGF, Vascular endothelial growth factor; ICAM-1, Adhesion molecule 1.

The parameter of the induced sequential vascular response was used to study several metabolite (sugars and L-histidine) and pharmacological (aspirin, insulin, and glucagon) challenges. There was also a correlation with a common oral glucose tolerance test. The findings showed a range of glucose, insulin, glucagon, L-histidine, and aspirin challenges produced both comparable and different behavioral responses (**Figure 4A**). New therapies might improve airflow through better regulation of vessel growth, dilatation, and leakage in the airway wall (110). One mechanism that endothelial cells act in asthma is by transendothelial migration (TEM). L-selectin and Intercellular Adhesion Molecule 1(*ICAM-1*)are critical TEM regulating genes, and animal asthma model lacking these cell adhesion molecules have decreased lung inflammation and lower airway hyperresponsiveness (AHR) in response to ovalbumin challenge (111–113), and allergic asthma patients also have increased endothelial adhesion molecules in bronchial biopsies (114). Elevated levels of adhesion molecules have been linked to asthmatics’ inflammation and underlying endothelial dysfunction (115, 116). Increased vascular permeability brought on by alterations to the blood-retinal barrier (BRB) is one of the primary effects of early diabetes (117). VE-cadherin proteolytic degradation has been suggested by observations as a possible mechanism by which diabetes promotes BRB deterioration (**Figure 4B**).

High glucose stimulated tissue transglutaminase 2 (*TG2*) expression and *TG2* silencing prevented Aβ-induced mitochondrial calcium influx, mtROS accumulation, and cell death in neuronal cells. In clinical treatment, the prevalence of tissue transglutaminase antibodies and diabetes with their first-degree relatives (118–122). Chemokine overexpression is one more potential explanation for the rise in asthma. In order to attract and activate circulating eosinophils, endothelial cells in asthma seem to release more chemokines (123). Mice with endothelial deficit of *TG2* display lower numbers of pulmonary eosinophils in response to allergen challenge, while pulmonary endothelium *TG2* is increased in asthma and seems to be necessary for eosinophil recruitment to the lung (124) (**Figure 4C**).

The neovascularization of the tracheal and bronchial arteries that develops in asthmatics with hyperreactive airways disease. This study’s findings were corroborated by bronchoscopy samples taken from the airways of asthmatic patients and healthy control volunteers, which revealed an increase in vessel size and density (125–127). By comparing the expression of *VEGF*, its receptors, and angiopoietin-1 in biopsy samples and bronchoalveolar lavage fluid between asthmatic sufferers and controls, researchers were able to better understand the processes causing this neovascularization (128). Endothelial markers as *CD31* or *VWF* used in histological analysis of parenchymal units could not distinguish between bronchial and pulmonary arteries. As a result, these models have been inaccurate in their appraisal of angiogenesis (129). Additionally elevated in asthmatics’ produced sputum samples, *VEGF* has a negative correlation with FEV1 (130). Additionally, several *VEGF* polymorphisms (such as rs4711750 and rs3025038) seem to enhance the chance of developing asthma and are linked to lung function (131).Further research is required to determine the processes behind the development of new vascular beds in adults. However, it is still challenging to distinguish the growth factors and molecular processes of lung angiogenesis from underlying diseases consequences (110, 130, 132–142) (**Figure 4D**).

GLP-1R is expressed in endothelial cells (143). The usage of GLP-1RAs in the management of T2DM is recommended. GLP-1RAs may also lessen airway inflammation and hyperresponsiveness at the same time. GLP-1RAs prevent adult asthmatics’ symptoms from deteriorating as much. GLP-1RAs could provide a fresh method for treating asthma brought by metabolic malfunction (144–149). As a result of increased expression of *DPP-4*, which is induced by the type 2 cytokines interleukin (IL)-4 and IL-13, in diseases of asthma and type 2 inflammation, such as atopic dermatitis and chronic rhinitis, DPP-4i may help with the management of asthma (103, 150–154). Glibenclamide (targeting sulfonylurea receptor 1) may lessen airway inflammation because it inhibits ATP-sensitive potassium (*KATP*) channels. Glibenclamide significantly decreased the AHR, airway inflammation, and T-helper type 2 (Th2) cytokines in a mouse model of asthma caused by ovalbumin (OVA). Additionally, Glibenclamide decreased the lung’s phosphorylated signal transducer and activator of transcription 6 (*p-STAT6*) and vascular cell adhesion molecule 1 (*VCAM-1*) expression that was brought on by OVA. These findings provide evidence for the safety of prescribing Glibenclamide in diabetic patients with comorbid asthma, as well as suggest a potential new therapeutic role for asthma through a pathway related to targeting the control medications for diabetes. These findings suggest that GLP-1R, *DPP-4*, and the sulfonylurea Glipalamides play an important role in the development of asthma (155–158) (**Figure 4E**). However, fewer studies have been undertaken on the combination of asthma and diabetes to yet, and endothelial cell dysfunction may become a prominent study area. Future research should focus more on this process to optimize the usage of medications and enhance patients’ lung function and prognosis to a greater extent.

### IPF and T2DM

As was already indicated, the lung has a dense network of connective tissue and alveolar capillaries, indicating that it can be one of the targets of diabetic microvascular damage. Recent research has shown that having high blood sugar levels might cause interstitial fibrotic alterations and alveolar microangiopathy (159, 160). The three main stages of alveolar microangiopathy, in which hyperglycemia may cause interstitial fibrosis, are: (1) oxidative stress injury, which initiates IPF; (2) alveolar inflammation, immune cell activation, secretion of numerous pro-inflammatory factors, which activates endothelial cells; and (3) pro-fibrotic cytokines secreted by endothelial cells, which mark the end of the disease (25, 161–163).

**Figure 5:**
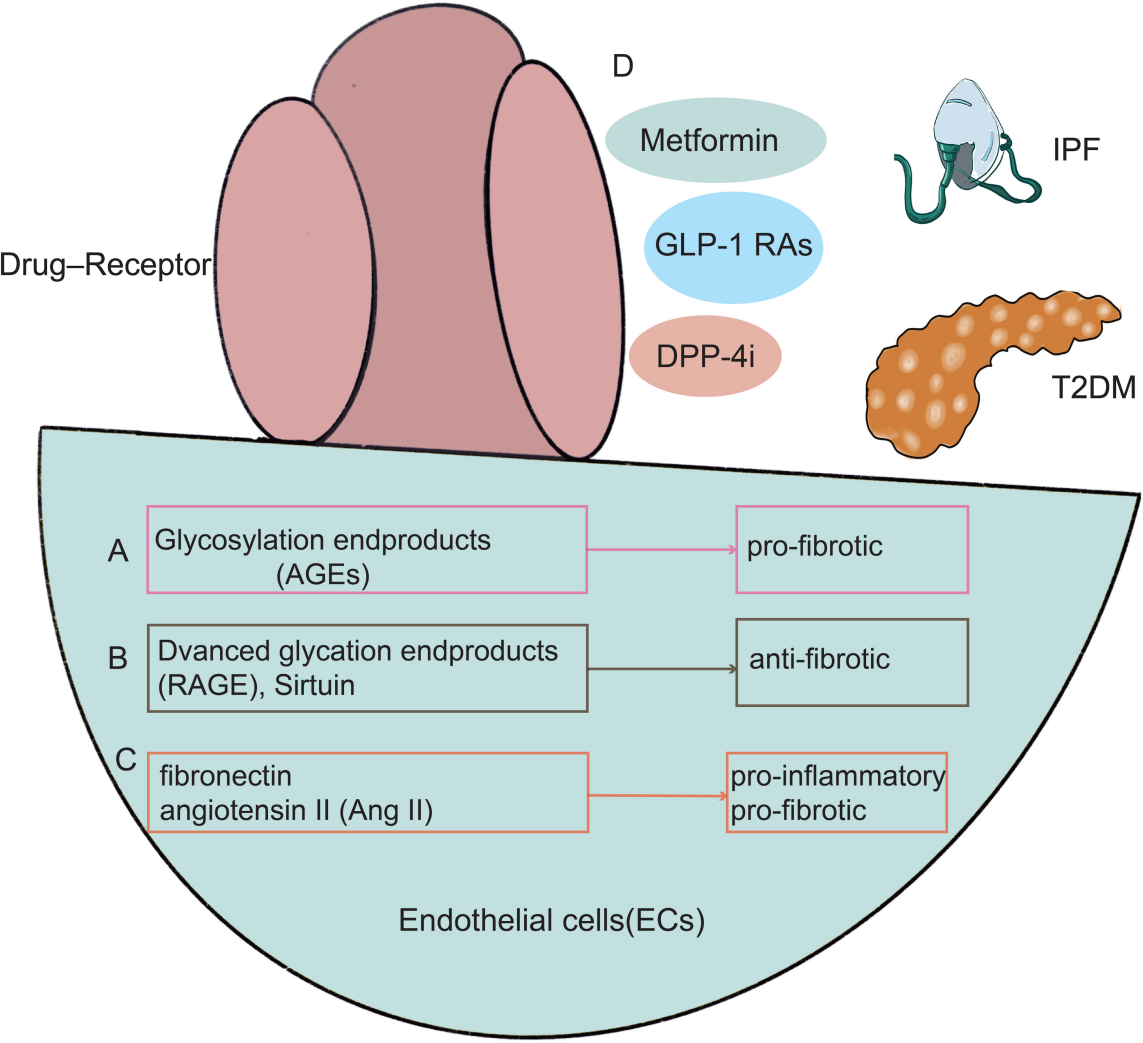
There are shared mechanisms in endothelial cell dysfunction in T2DM with IPF, with existing research focused on AGEs **(A)**, RAGE **(B)**, and Ang II **(C)**. In the meanwhile, diabetic therapies such as metformin, GLP-1 receptor agonists, DPP-4 inhibitors, and PPAR-agonists may alleviate pulmonary function decline by enhancing endothelial function **(D)**. GLP-1 RAs, GLP-1 receptor agonists; DPP-4i, Dipeptidyl peptidase 4 inhibitors.

The common pathways of endothelium damage-related lung injury in diabetes are listed below. Endothelial cells exhibit higher levels of expression of *AGEs, RAGE*, and sirtuin (*SIRT*), which are also changed in diabetes and are strongly linked to the onset of the disease (164, 165) (**Figures 5A, B**). Under typical circumstances, diabetes causes *AGEs* to accumulate in the lungs (166). Researchers discovered significant elevated *AGEs* in diabetic patients, which resulted in pulmonary fibroblast aggregation (167). *RAGE* is thought to stimulate the production of *TGF-β* and to inhibit the activation of *Smad2*, *ERK*, and *JNK* signaling (168, 169). *TGF-β* (170) is a key player in tissue remodeling and fibrosis and is a mediator of pro-fibrotic characteristics. *SIRT* has a possible involvement in the treatment of IPF by mediating the processes of insulin secretion, cell cycle, and apoptosis (171, 172). *SIRT* is crucial in the development of diabetic microangiopathy (173, 174). *SIRT* blocks oxidative stress, the *IL-1*, and *TGF-1/Smad3* signaling pathways, which are pro-inflammatory cytokines, mitochondrial DNA damage, and fibronectin. Additionally, pro-inflammatory or pro-fibrotic substances, including fibronectin, angiotensin II (Ang II) that are heavily implicated in the development of the diabetic lung were also shown to be elevated in the lung tissue of diabetic mice (175) (**Figure 5C**). As a consequence of several pathways being triggered in a high-glucose environment, intracellular stress and abnormal cytokine production occur. Damage to the structural lungs and pathological pulmonary fibrosis result from the failure of re-endothelialization and consequent loss of the alveolar-capillary barrier basal layer’s integrity.

In the lungs of IPF patients, metformin has potent antifibrotic effects *via* metabolic pathway modulation, inhibition of *TGF-1* activity, inhibition of collagen synthesis, activation of *PPAR* signaling, and induction of lipogenic differentiation of fibroblasts (176–181). By activating *AMPK* and enhancing the *TGF*- signaling pathway, metformin induces the inactivation and apoptosis of myofibroblasts, reversing the progression of pulmonary fibrosis (180). Gu et al. studied that metformin-activated *AMPK* can downregulate Forkhead Box M1 (*FOXM1*) and alleviate BLM-induced IPF model in mice (177). *GLP-1* is a crucial hormone that regulates hunger, glucose metabolism, and insulin secretion. A 39 amino acid agonist of *GLP-1*receptor, exendin-4. By deactivating *NF-κB*, *GLP-1* receptor agonists greatly reduce bleomycin (BLM)-induced lung damage and fibrosis in mice (182).Exendin-4, meanwhile, reduced hyperglycemia-related lung damage by lowering oxidative stress and promoting cell growth (183).*DPP-4* inhibitors and *PPAR-γ* agonists have proven useful in the treatment of pulmonary fibrosis. In a mouse model of pulmonary fibrosis after systemic endotoxin damage, endothelial to mesenchymal transition (EndMT) was shown in endothelial cells overexpressing *DPP-4*(Vildagliptin), which has been reported to alleviate pulmonary fibrosis. Vildagliptin, which inhibits EndMT, may be helpful in treating pulmonary fibrosis (184). *PPAR*- is a member of the nuclear hormone receptor superfamily, and its effects include modifying metabolic and inflammatory responses, among others. Recent research has also shown the effectiveness of *PPAR*- agonists in treating BLM-induced lung fibrosis (185, 186). In a mouse model, rosiglitazone and selegiline inhibited *TGF-1*-mediated fibrotic alterations in alveolar epithelial EMT, differentiation of myoblasts, and collagen synthesis, indicating a therapeutic efficacy of *PPAR*- ligands in fibrotic lung injury (187, 188) (**Figure 5D**).

In conclusion, endothelial cells are critical in the development of pulmonary fibrosis brought on by hyperglycemia. Additionally, endothelial cells must be the primary focus of therapeutic intervention; thus, determining whether endothelial cells are involved in the regulation of lung fibers might be a promising therapeutic avenue for the investigation of diabetes-related pulmonary fibrosis.

## Conclusion

Future studies should focus on the mechanism and clinical management of respiratory diseases and diabetes, especially due to the COVID-19 pandemic when severe lung consequences. It is possible to improve the treatment of patients with diabetes and pulmonary diseases by understanding the interactions between the two disorders and developing an effective treatment strategy. In addition, further research is essential to pave the way for potential treatments by using endothelial cells in discussions of co-morbidities underlying mechanisms.

## Author contributions

LZ, FJ, and CL, conception or design of the work and critical revision of the article. LZ, YX, and YM, drafting the article and data collection. XZ, drafting the manuscript and revision of the article. All authors contributed to the article and approved the submitted version.
